# Effects of high-fidelity simulation education on medical students’ anxiety and confidence

**DOI:** 10.1371/journal.pone.0251078

**Published:** 2021-05-13

**Authors:** Ji Hye Yu, Hye Jin Chang, Soon Sun Kim, Ji Eun Park, Wou Young Chung, Su Kyung Lee, Miran Kim, Jang Hoon Lee, Yun Jung Jung

**Affiliations:** 1 Office of Medical Education, Ajou University School of Medicine, Suwon, South Korea; 2 Department of Obstetrics & Gynecology, Ajou University School of Medicine, Suwon, South Korea; 3 Department of Gastroenterology, Ajou University School of Medicine, Suwon, South Korea; 4 Department of Pulmonary and Critical Care Medicine, Ajou University School of Medicine, Suwon, South Korea; 5 Ajou Center for Clinical Excellence, Ajou University School of Medicine, Suwon, South Korea; 6 Department of Pediatrics, Ajou University School of Medicine, Suwon, South Korea; Uniformed Services University of the Health Sciences, UNITED STATES

## Abstract

**Introduction:**

Psychological factors such as anxiety and confidence that students have in the patient care situation are important in that this affects the actual clinical performance. Students who are just starting clinical practice have a lack of clinical knowledge, skill proficiency, and patient communication skills, so they experience anxiety and lack of confidence in clinical setting. Practice in a safe environment, such as simulation education, can help students perform more settled and competently in patient care. The purpose of this study was to analyze the effect of high-fidelity simulation experience on anxiety and confidence in medical students.

**Materials and methods:**

This study enrolled 37 5^th^-year students at Ajou University School of Medicine in 2020. Two simulation trainings were implemented, and a survey was conducted to measure students’ level of anxiety and confidence before and after each simulation. Based on the research data, a paired t-test was conducted to compare these variables before and after the simulation, and whether this was their first or second simulation experience.

**Results:**

Students had a significantly lower level of anxiety and a significantly higher level of confidence after the simulation than before. In addition, after one simulation experience, students had less anxiety and more confidence before the second simulation compared to those without simulation experience.

**Conclusions:**

We confirmed that medical students need to be repeatedly exposed to simulation education experiences in order to have a sense of psychological stability and to competently deliver medical treatment in a clinical setting. There is a practical limitation in that medical students do not have enough opportunities to meet the patients during clinical practice in hospitals. Therefore, in order to produce excellent doctors, students should have the expanded opportunities to experience simulation education so they can experience real-world medical conditions.

## Introduction

Medical students entering the course of clinical practice have anxiety related to clinical practice [1]. Medical students at the beginning of clinical practice often do not have sufficient skills in history-taking, physical examination, and diagnosis [2]. Therefore, students experience a lack of confidence in their diagnostic and therapeutic skills during clinical practice, and they suffer from psychological distress such as stress and anxiety caused by the responsibility in treating patients [3, 4]. According to a prior study [5], students lacked confidence in patient care skills, and this was the most contributing factor to mental distress in clinical practice. At the beginning of clinical practice, students may experience anxiety and lack of confidence in the patient care situation due to lack of clinical knowledge, technical proficiency, and patient communication skills.

Psychological factors such as anxiety and confidence are important because they affect actual clinical performance. High levels of anxiety affect the ability to perform treatment, increase the likelihood of making mistakes, and have a negative impact on the success of clinical practice [6, 7]. Confidence in itself does not directly affect clinical performance, but rather through anxiety [7]. Anxiety mediated the relationship between confidence and clinical performance. In other words, low confidence increases anxiety and disturbs students from performing properly in clinical situations.

It is necessary to improve students’ patient care competence through various clinical experiences in a safe environment. Students with abundant medical experience show confidence in patient care [8], and students with high self-confidence also show improvement in clinical skills [9]. In other words, the medical student’s confidence increases as they gain experience [10], and an increase in confidence indicates an improvement in competency [7]. For medical students who have limited opportunities to face real patients, it is necessary to provide an environment similar to real clinical situations to help students better prepare.

Simulation education provides an environment very similar to the clinical field so that students can experience practical clinical treatment [11]. Simulation is a new teaching model and is used widely in medical education [12]. Simulation-based education allows students to experience low-frequency or high-risk scenarios, and to improve their knowledge, skills, and attitudes [13]. Simulation education can also be used as an alternative solution to problems such as lack of confidence among students in performing actual patient care [14]. In particular, high-fidelity simulation is emerging as an effective educational method to develop confidence in clinical practice [15]. In the present situation, in which hospital practice is impossible or limited due to the spread of infectious diseases such as COVID-19, there are reduced opportunities for medical students to see patients directly. In this case, high-fidelity simulation-based education can be actively utilized as an alternative method to equip students with medical competence through clinical exposure. If simulation education provides medical students with a variety of treatment experiences, students feel less anxiety and increase their confidence in patient treatment situations; this improves their clinical competence. Several studies have reported that different forms of simulation education conducted on Healthcare students improve student confidence [16]. Also, the anxiety that occurs in a particular situation or stimulus can be reduced through repeated exposure to it [17, 18]. Therefore, this study aims to identify the high-fidelity simulation experience, especially how repeated exposure to simulations, affects the level of anxiety, confidence in medical school students.

The research questions of this study are as follows.

1. Are there any differences in the levels of anxiety and confidence among students before and after simulation-based education experience?
2. Is there a difference between the stated anxiety and confidence level of students before education, depending on their previous experience in simulation-based education?

## Materials and methods

### Participants

40 students enrolled in the 5th grade of Ajou University School of Medicine participated in the simulation education program in 2020. We excluded data from three people whose missing values occurred due to missing responses to some of the questions in the survey for this study. A total of 37 people (male: 26, female: 11) who responded to the questionnaire for this study provided research data. The mean (M) of the age of study participants was 24.34 (standard deviation, SD = 1.33) years.

### Ethical considerations

This study was approved by the Institutional Review Board (IRB) of Ajou University Hospital (Ethics consent No. AJIRB-SBR-SUR-20-255). It proceeded with consent exemption. This study was conducted to confirm the educational effect of the high-fidelity simulation education implemented as a part of the regular clinical clerkship program. The analysis was conducted through retrospective research based on the survey data received from the participating students, so consent was exempted.

### Setting

Students experienced clinical practice for the first time in their fifth year of medicine. From April 10 to June 5, 2020, students were divided into six groups and rotated in nine internal medicine departments every week. High-fidelity simulators (Laerdal’s SimMan patient simulator) were used to simulate patient cases during clinical practice in pulmonary and gastroenterology. Students experienced two simulation training sessions during the 9-week training period. The first and second simulations of the four groups were conducted over one week, and two groups had 2-week and 6-week terms. Each group includes 6 to 7 students, and when performing the simulation, each group divided into one small group of two to three students to conduct the simulation. And the student group was composed differently in each of the two simulations. Each group was randomly assigned one scenario out of six pulmonary and seven gastrointestinal disease scenarios, and students experienced one each case of pulmonary disease and gastrointestinal disease. Students distributed tasks within the group according to the scenario and performed their assigned tasks accordingly. The pulmonary disease scenarios were for symptoms of acute respiratory distress and consisted of pneumonia with parapneumonic effusion, COPD with acute exacerbation, pulmonary embolism, heart failure with pulmonary edema, spontaneous pneumothorax, and iatrogenic tension pneumothorax. The gastrointestinal scenarios included melena, jaundice, hematemesis, diarrhea, abdominal pain, abdominal distension, and abdominal mass. During the simulation for each scenario, data such as vital signs, radiographs, electrocardiograms, and laboratory results were presented when requested by the student. The required procedures for students during simulation are history-taking, physical examination and vital signs measurement, differential diagnosis, treatment or treatment planning according to diagnosis. The learning objectives of each simulation focused on students being able to conduct initial assessment, diagnosis, and treatment plans through history taking and physical examination, and effective teamwork and communication with the patient.

### Procedures

**Fig 1:**
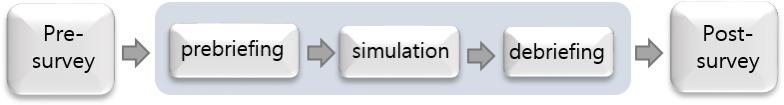
Simulation design.

The simulation training session was conducted in the order presented in Fig 1. During prebriefing before simulation, the students identified the necessary equipment during the procedure, distributed tasks within the group, and prepared to perform according to the scenario, such as checking the data on the main symptoms and current medical history of the provided patient. The simulation was then carried out for 10–15 minutes. Each simulation was supervised by one faculty, and feedback was provided in terms of knowledge, skill, and attitude about the good and bad points found while observing the performance of each group. In addition, a pre- and post-survey was conducted to compare the effect of simulation education.

### Measures

The short-form of the state scale of the Spielberger State-Trait Anxiety Inventory (STAI) developed by Marteau & Bekker [19] was used to measure anxiety. It consists of a total of 6 questions; however, in this study, a total of 5 questions were used, excluding ’I feel upset’, which lowers the internal consistency between questions and does not correlate significantly with other items. In this study, each item was measured on a 4-point Likert scale from ’totally disagree’ (1 point) to ’totally agree’ (4 points), and positive items (calm, relaxed, content) were reverse scored [20, 21]. To calculate the total score, all five scores were summed, and then the total score was multiplied by 20/6. In this study, the Cronbach α of anxiety was .839 before the first simulation, .728 after the first simulation, .752 before the second simulation, and .809 after the second simulation.

Among the simulation survey questions developed by Cato [22], 11 items suitable for this study were used to measure the students’ confidence in simulation performance. This consisted of one question before the simulation and 10 questions during the simulation. Each item was measured on 5-point Likert scale from ’very confident’ (1 point) to ’very anxious’ (5 points). The range of confidence scores in the pre-simulation question was 1–5 points, and the range of total scores in during simulation situation was 10–50 points. All items were reversely coded, so the higher the total score, the higher the confidence. In this study, the Cronbach α was 0.931 before the first simulation and .923 after, and it was 0.904 before the second simulation and 0.857 after.

### Statistical analysis

Data that evaluated students’ anxiety and confidence before and after the simulation experience using the high-fidelity simulator were used for analysis. Before and after the simulation, a paired t-test was performed to compare the difference in the scores for each variable based on the presence of prior simulation experience.

## Results

Table 1 shows a comparison of changes in anxiety levels according to simulation practice. In both the first and second simulation, anxiety decreased after the simulation (M = 45.41, SD = 12.12; M = 43.14, SD = 12.04) compared to before (M = 61.41, SD = 12.01; M = 56.86, SD = 11.36). Students’ anxiety before the practice can be seen as a result of the psychological tension related to their performance in a simulation situation. We then compared differences in anxiety levels based on whether students had previous simulation practice experience, and found that pre-simulation anxiety levels were significantly lower in second simulation (M = 56.86, SD = 11.36) compared to the first simulation (M = 61.41, SD = 12.01) (t = 2.78, *p*<0.01).

**Table 1:** Comparison of changes in anxiety levels according to simulation practice.

Item			Mean	Standard deviation	t (pre-post)	t (pre1-pre2)
1. I feel calm*	1^st^	Pre	3.19	0.78	4.56***	2.19*
		Post	2.35	0.79		
	2^nd^	Pre	2.84	1.01	3.29**	
		Post	2.16	0.76		
2. I am tense	1^st^	Pre	3.05	0.70	5.74***	1.86
		Post	1.95	0.88		
	2^nd^	Pre	2.81	0.70	5.79***	
		Post	1.92	0.72		
3. I am relaxed*	1^st^	Pre	3.24	0.64	4.38***	2.52*
		Post	2.43	0.87		
	2^nd^	Pre	2.97	0.76	3.85***	
		Post	2.35	0.79		
4. I feel content*	1^st^	Pre	2.76	0.83	0.65	-0.26
		Post	2.62	1.04		
	2^nd^	Pre	2.78	0.71	1.98	
		Post	2.41	0.86		
5. I am worried	1^st^	Pre	3.11	0.88	5.74***	2.14*
		Post	2.00	0.78		
	2^nd^	Pre	2.81	0.78	4.74***	
		Post	1.95	0.85		
Total	1^st^	Pre	61.41	12.01	5.39***	2.78**
		Post	45.41	12.12		
	2^nd^	Pre	56.86	11.36	5.0***	
		Post	43.14	12.04		

pre1: 1^st^ pre score, pre2: 2^nd^ pre score

*p < 0.05

**p < 0.01

***p < 0.001

Comparison of changes in levels of confidence according to simulation practice is given in Table 2. In both the first and second sessions, students felt more confident post-simulation (M = 31.92, SD = 7.25; M = 32.51, SD = 4.89) compared to pre-simulation (M = 26.59, SD = 6.93; M = 29.76, SD = 5.81). In addition, Pre-simulation confidence was significantly higher in the second simulation (M = 29.76; SD = 5.81) compared to the first simulation (M = 26.59, SD = 6.93) (t = -3.57, p<0.01).

**Table 2:** Comparison of changes in confidence level according to simulation practice.

	Item			Mean	Standard deviation	t (pre-post)	t (pre1-pre2)
Before simulation	1. The preparation before simulation contributes to my feeling_	1^st^	Pre	2.38	1.01	-	-2.49*
		2^nd^	Pre	2.76	0.80	-	
During simulation	2. Caring for a patient in the simulation room environment contributes to my feeling_	1^st^	Pre	2.57	1.01	-2.39*	-1.60
			Post	3.03	0.99		
		2^nd^	Pre	2.78	0.79	-4.06***	
			Post	3.22	0.71		
	3. Working with the medical equipment in the simulation room contributes to my feeling_	1^st^	Pre	2.32	0.85	-3.71**	-2.75**
			Post	2.89	1.13		
		2^nd^	Pre	2.73	0.96	-1.96	
			Post	3.00	0.75		
	4. Distinguishing between what is real and what is simulated(like patient assessment data or operation of equipment) contributes to my feeling_	1^st^	Pre	2.51	0.84	-2.99**	-3.16**
			Post	3.03	0.99		
		2^nd^	Pre	3.08	0.89	-1.22	
			Post	3.22	0.71		
	5. When working with the mannequin I feel_	1^st^	Pre	2.86	0.79	-2.14*	-1.53
			Post	3.16	0.90		
		2^nd^	Pre	3.03	0.73	-2.58*	
			Post	3.32	0.75		
	6. Being “on camera” contributes to my feeling_	1^st^	Pre	2.51	0.93	-5.77***	-2.19*
			Post	3.22	0.85		
		2^nd^	Pre	2.86	0.98	-2.23*	
			Post	3.19	0.70		
	7. Caring for a patient with my team contributes to my feeling_	1^st^	Pre	3.51	0.84	-1.28	0.40
			Post	3.73	0.93		
		2^nd^	Pre	3.46	0.69	-1.87-	
			Post	3.65	0.75		
	8. When making a decision about the patient I feel_	1^st^	Pre	2.84	0.87	-4.09***	-1.86
			Post	3.46	0.90		
		2^nd^	Pre	3.08	0.76	-2.49*	
			Post	3.43	0.69		
	9. Performing in front of my peers contributes to my feeling_	1^st^	Pre	2.70	0.91	-3.53**	-1.97
			Post	3.24	0.83		
		2^nd^	Pre	3.03	0.73	-3.09**	
			Post	3.43	0.73		
	10. Performing in front of faculty contributes to my feeling_	1^st^	Pre	2.35	0.98	-4.70***	-3.40**
			Post	3.05	0.91		
		2^nd^	Pre	2.92	0.83	0131	
			Post	3.11	0.77		
	11. The possibility of making a mistake contributes to my feeling_	1^st^	Pre	2.41	0.98	-4.87***	-2.67*
			Post	3.11	0.99		
		2^nd^	Pre	2.78	0.79	-1.23	
			Post	2.95	0.81		
	Total	1^st^	Pre	26.59	6.93	-5.22***	-3.57**
			Post	31.92	7.25		
		2^nd^	Pre	29.76	5.81	-3.82**	
			Post	32.51	4.89		

pre1: 1^st^ pre score, pre2: 2^nd^ pre score

*p < 0.05

**p < 0.01

***p < 0.001

## Discussion

When comparing students’ anxiety and confidence pre- and post-simulation scores showed that anxiety decreased and confidence increased significantly after the simulation. We interpret this to mean that the levels of anxiety were lowered after the practice, although students’ anxiety increased in the tense situation before the practice. A prior study found that high-fidelity human patient simulation experience reduces students’ anxiety about communicating with patients in a real patient encounter [23]. It was also reported that students who experienced high-fidelity simulation education had lower preclinical anxiety than those who did not [24]. In this study, the improvements in students’ confidence after simulation suggest that students had confidence in their overall management in the simulation situation through simulation experience. The analysis of the impact of simulation training on participant confidence showed mixed results [25, 26]. We expect that simulation allows students to practice treatment with more confidence in patient interaction. However, students may become not overconfident as they evaluate their own performance more objectively by experiencing the procedure of the practice directly in a similar situation. Whether simulation education makes students more confident, simulation experiences have a sufficient educational effect.

There were significant differences in students’ anxiety and confidence before simulation practice between no simulation experience and after one experience. Students’ anxiety before the simulation was lower and confidence was higher after the simulation experience, compared to no previous simulation experience. It can be inferred that as the simulation experience accumulates, students will be able to face medical situations with more confidence and psychological stability. As previously demonstrated, repeated exposure to high-fidelity simulation experience lowered students’ negative emotions such as anxiety and depression about patient care and improved confidence in performance [27, 28]. Research has shown that exposure to real-like virtual situations is effective in lowering anxiety and providing psychological stability [29, 30]. Thus, continuous exposure to simulation practice helps students to improve their clinical competency by reducing anxiety and enabling them to perform skills and communicate with patients more confidently.

This study confirmed the effect of high-fidelity simulation in medical education through empirical research. In particular, we confirmed that simulation education can affect anxiety and confidence in clinical performance, and improve psychological stability and clinical efficacy in medical treatment. This has confirmed the importance of clinical practice education using high-fidelity simulation in improving the clinical competency of physicians, which is directly connected to patient safety. In particular, the subjects of this study had limited clinical practice in order to prevent the spread of COVID-19. Therefore, they may not properly experience treatment procedures for patients in the ward, and this could cause problems due to lack of understanding patient treatment cases. In this situation, high-fidelity simulation education was a good alternative method that allowed students to simulate real medical practice, and it is very meaningful to confirm the educational effect of this method.

The limitations of this study and suggestions for follow-up studies are as follows.

This study was limited in that anxiety and confidence were measured only through students’ self-reported data about their psychological state using a standardized questionnaire tool. This study also has limitations in that it does not contain actual performance measurements as well as psychological variables such as anxiety and confidence. More objective measurements of anxiety and confidence would be supplemented by data that measures the level of anxiety using physical indicators and objective performance assessments by evaluators. In addition, the data analysis here was based on only two simulation experiences, and the two simulation training sessions were not conducted equally for all groups. Scenarios of the same subject were not executed in the same order and at the same intervals. That is, the participants are not all equivalent in terms of their ability to provoke anxiety. The present study also has limitations in that the insufficient number of participants made it difficult to analyze differences between groups according the level of anxiety and confidence. In the future, follow-up of simulation training programs at regular intervals and subsequent measurement of research variables could better explain the effects of repeated exposure to simulation training. And if a subsequent study with more lager number of participants is conducted, analyzing inter-group differences based on the characteristics of learners related to anxiety and confidence may provide important educational implications.

## Conclusions

This study was conducted to confirm the effect of high-fidelity simulation-based education on anxiety and confidence in medical students. After the simulation education, students’ anxiety decreased and their confidence improved. In addition, Students had less anxiety and more confidence before the second simulation compared to the first simulation. Therefore, we have confirmed that medical students need to be repeatedly exposed to simulation education experiences in order to have a sense of psychological stability and perform with confidence in a clinical setting. There is a practical limitation that medical students are not provided with sufficient conditions to actually experience medical practice in the course of their clerkships. Therefore, opportunities for high-fidelity simulation education that can provide experience similar to actual conditions should be expanded in order to produce doctors with excellent medical competency.

## Supporting information

S1 Dataset (SAV)
